# Enhancing structural complexity: An experiment conducted in the Black Forest National Park, Germany

**DOI:** 10.1002/ece3.9732

**Published:** 2023-01-09

**Authors:** Thomas Asbeck, Adam Benneter, Alexander Huber, Damaris Margaritis, Jörn Buse, Flavius Popa, Patrick Pyttel, Marc Förschler, Stefanie Gärtner, Jürgen Bauhus

**Affiliations:** ^1^ Chair of Silviculture University of Freiburg Freiburg Germany; ^2^ Department of Ecological Monitoring, Research and Species Conservation Black Forest National Park Freudenstadt Germany; ^3^ Unique land use GmbH Freiburg Germany

**Keywords:** conservation area management, forest structure, morticulture, *Picea abies*, tree‐related microhabitats

## Abstract

We report on a structural complexity enhancement (SCE) experiment that was designed to test ecological restoration measures in the Black Forest National Park, Germany. The main goal was to understand as to whether the creation of standing and downed deadwood within previously managed, single‐layered Norway spruce (*Picea abies* L.) forests accelerates the development of forest structure, richness, and diversity of a range of taxonomic groups. Here we introduce the experimental design and describe the development of stand structure including abundance and richness of tree‐related microhabitats (TreMs) within 5 years after initiation of the experiment in October 2016. To enhance structural complexity in treatment plots, 10 trees per plot were toppled using a skidder winch, and another 10 trees were ring barked at a height of around 60 cm above ground level with a chainsaw. To monitor stand structure, we collected data on common forest attributes such as diameter at breast height (DBH), tree height, and TreMs of all trees in the six experimental and six control plots measuring 0.25 ha in size before the treatments were carried out in 2016 and again in 2020/21. We analyzed the abundance and richness of TreMs using generalized linear mixed models with DBH and treatment vs. control as predictors. The SCE treatment resulted in a significant increase in deadwood volumes (4.2 vs. 439.5 m^3^) as well as in TreM abundance and richness (increase of 0.74 TreMs per tree). This indicates that the SCE treatment was effective to increase biodiversity‐relevant structures such as deadwood and TreMs, in previously managed Norway spruce‐dominated stands. The ongoing monitoring of a range of taxonomic groups (birds, bats, small mammals, coleoptera, fungi, mosses, and vascular plants) in this experiment will demonstrate to what extent the enhancement in structural complexity will lead to an enrichment in species richness and diversity.

## Graphical abstract

Here we report about the forest structural changes brought about by a strcutural complexity enhancement approach in secondary spruce stands in the Black Forest National Park. Five years after the initial treatment (toppling and ring‐barking of some trees), the dead wood volume and tree‐related microhabitats had significantly increased in the treated plots. Subsequent studies will show how this treatment has influenced species richness and diversity of a range of taxonomic groups.

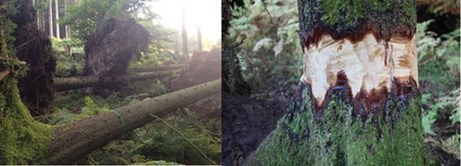

## 1 INTRODUCTION

In recent times, there have been considerable advances in understanding the relation between structural complexity and forest biodiversity (Asbeck, Sabatini, et al., 2021; Schall, Schulze, et al., 2018). In many cases, the abundance of different structural elements and especially structures typically found in old‐growth forests are positively related to biological diversity metrics (Bauhus et al., 2009; Dove & Keeton, 2015; Gustafsson et al., 2012; Keeton, 2006; McKenny et al., 2006). One of the key factors for this effect appears to be that structurally complex forests offer both a higher number and diversity of habitat structures, in particular if they contain dead wood and very large trees (Basile et al., 2020; Hilmers et al., 2018; Paillet et al., 2018). Accordingly, most forests, especially in Europe and other regions where management intensity is relatively high, are typically less structurally complex than they would be without such management (Asbeck & Frey, 2021; Paillet et al., 2010; Schall, Gossner, et al., 2018). However, multi‐purpose continuous cover forest with late successional tree species mixtures has been a part of what farmers have been practicing for centuries in mountainous regions of Europe. Additionally, forest conversion from monospecific even‐aged conifer forests toward a more natural and site adapted tree species composition has been practiced in Central Europe including the Black Forest for decades (Yousefpour and Hanewinkel 2009). This shift toward more close‐to‐nature forestry was supported by society following acid‐rain‐related forest damages in the 1980 and severe wind storms followed by bark beetle outbreaks in the 1990s.

Despite these changes, forestry for wood production still focuses on relatively young stages of the forest successional cycle which lack of old growth structures (Asbeck et al., 2019; Hilmers et al., 2018). Although large elements of standing and downed deadwood are disproportionally important as habitat for forest‐dwelling species (Bauhus et al., 2018), they have accumulated in multiple‐use forests only at a slow rate for safety reasons and to avoid reductions in production capacity (Thorn, Chao, et al., 2020). This management history poses a problem when the management goals change toward a focus on promoting biodiversity and species conservation in forests. Such a paradigm shift is most evident when forests, used previously for wood production, are converted to strict reserves, as is the case in many development national parks such as the Black Forest National Park, Baden‐Württemberg, Germany. Large areas of this park are dominated by mono‐specific Norway spruce (*Picea abies* L.) forests that were managed for wood production prior to the parks' establishment in 2014. Invaluable experience with such a shift in management goals has been gathered in the Bavarian Forest National Park, Germany's first national park, which was also dominated by secondary Norway spruce forests at the time of its inception. There, refraining from active management to prevent the spread of bark beetles following storm damage to the Norway spruce forests in the park's core zone, led to the dieback of spruce stands at the scale of 37 square kilometers of the park (Heurich, 2001). Despite the widespread motivation to convert mono‐specific, not site‐adapted spruce forests including those still being managed for wood production, a radical “hands‐off” approach is, owing to the high risks of windthrow and bark beetles, in most cases not an option in central Europe (Seidel et al., 2019). This raises the question as to whether it is possible to employ an active restoration approach to accelerate the development towards old‐growthness, structural diversity and enhanced species diversity by using artificial disturbances that create standing and downed dead wood as well as canopy gaps in secondary spruce forests (Bauhus et al., 2009; Gustafsson et al., 2020; Koivula & Vanha‐Majamaa, 2020; Sandström et al., 2019). If this approach leads to mixed species and uneven‐aged forests, it may also reduce the probability of large scale, stand replacing disturbances that are typical for even‐aged spruce forests (Albrich et al., 2020).

The study presented here was carried out as a forest restoration research experiment within the development zone of the Black Forest National Park. This zone is destined to be added to the strictly protected core area of the national park, which is without forest management interventions, within the first 30 years since establishment. The aim of the experiment was to determine whether biodiversity could be enhanced by creating structural complexity by increasing the amounts of standing and downed deadwood within even‐aged Norway spruce dominated forests. For that purpose, the forest structure including tree‐related microhabitats (TreMs) were inventoried before the creation of dead wood and canopy gaps in early‐mature (80–90 years old) spruce‐dominated forest that were conventionally managed before the establishment of the national park in 2014. One key aspect to keep in mind during the creation of structurally more complex forests is the provisioning of TreMs. These structures provide important habitats for forest‐dwelling species and are defined as “distinct, well delineated structures occurring on living or standing dead trees, that constitute a particular and essential substrate or life site for species or species communities during at least a part of their life cycle to develop, feed, shelter or breed” (Larrieu et al., 2018). These structures have been reported to increase with tree diameter at breast height (DBH) and are often more abundant in broadleaved trees than in conifers and differ between dead and live trees (Paillet et al., 2017; Spinu et al., 2022) and in primary compared to managed forests (Asbeck, Kozák, et al., 2021). To our knowledge, this is the first structural complexity enhancement (SCE) experiment done in secondary spruce forests that also addressed the dynamics of TreMs.

In our study, we addressed the following research questions:

- How did forest structure in terms of volumes in living trees and deadwood respond to the SCE treatments?
- Did the experiment influence the abundance and richness of tree‐related microhabitats?

## 2 MATERIALS AND METHODS

### 2.1 Experimental sites and data collection

Our study plots were situated in a spruce‐dominated mountain forest within the development zone of the Black Forest National Park, approximately 30 km north of Freudenstadt in the state of Baden‐Württemberg, Germany. The bedrock in the study area consists mostly of granite, bordered by a sandstone stratum at the northern and uphill edge of the experimental site. Soils range from gleys in the west to podsol‐brown soils in the east. The area is characterized by relatively high mean annual precipitation (long‐term annual mean 1787 mm for the reference period 1982–2010) and moderate mean annual temperatures (long‐term annual mean 8.1°C for the same period), with a vegetation period lasting from March to October with 1073 mm of precipitation during this period.

**FIGURE 1:**
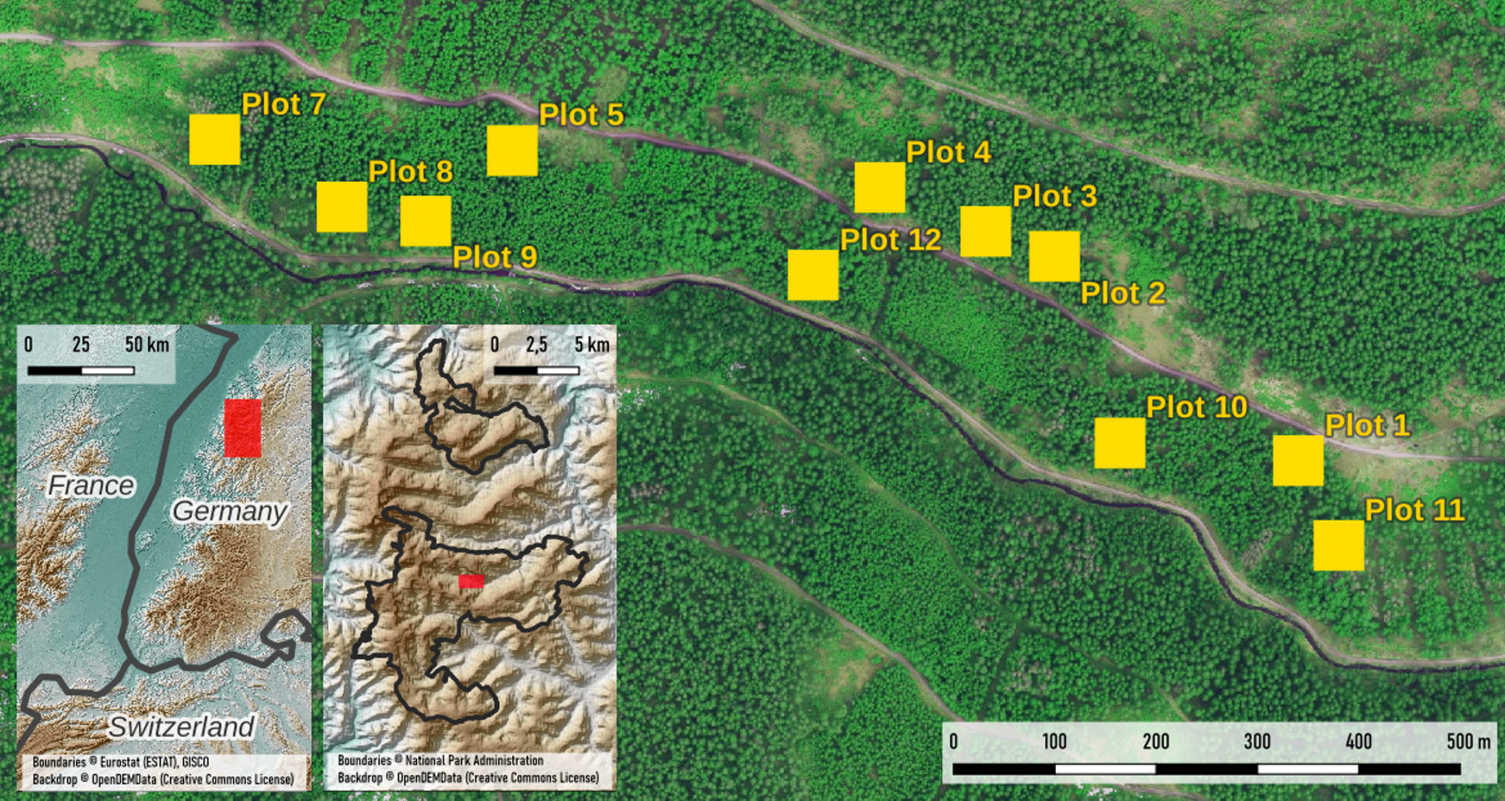
Location of the Black Forest National Park in South‐Western Germany and the location of the experiment and its plots in the Schönmünztal in the southern section of the National Park. Plots 1, 3, 6, 7, 8, and 10 received the SCE treatment.

For the experiment, we used a before‐after‐control‐impact (BACI) design. For that purpose, we selected 12 study plots situated in the valley “Schönmünztal” (GPS: 48.56604, 8.28657) in the national park (Figure 1). Six of these plots were treated to enhance structural complexity, whereas the other six plots were controls. The experiment was located in terrain with slopes ranging from flat to 35° (mean: 19.7°). Between the plots, a distance of at least 30 meters was kept as a buffer zone and to avoid or reduce spatial autocorrelation. The plots were selected after a comprehensive screening of the study area for patches with early‐mature (80–90 years old), even‐aged, Norway spruce‐dominated stands with a closed canopy. One corner of the 50 × 50 m plots was marked as the 0‐corner and measured with a GPS device. Subsequently, the azimuths of all sides of each plot were also recorded.

**FIGURE 2:**
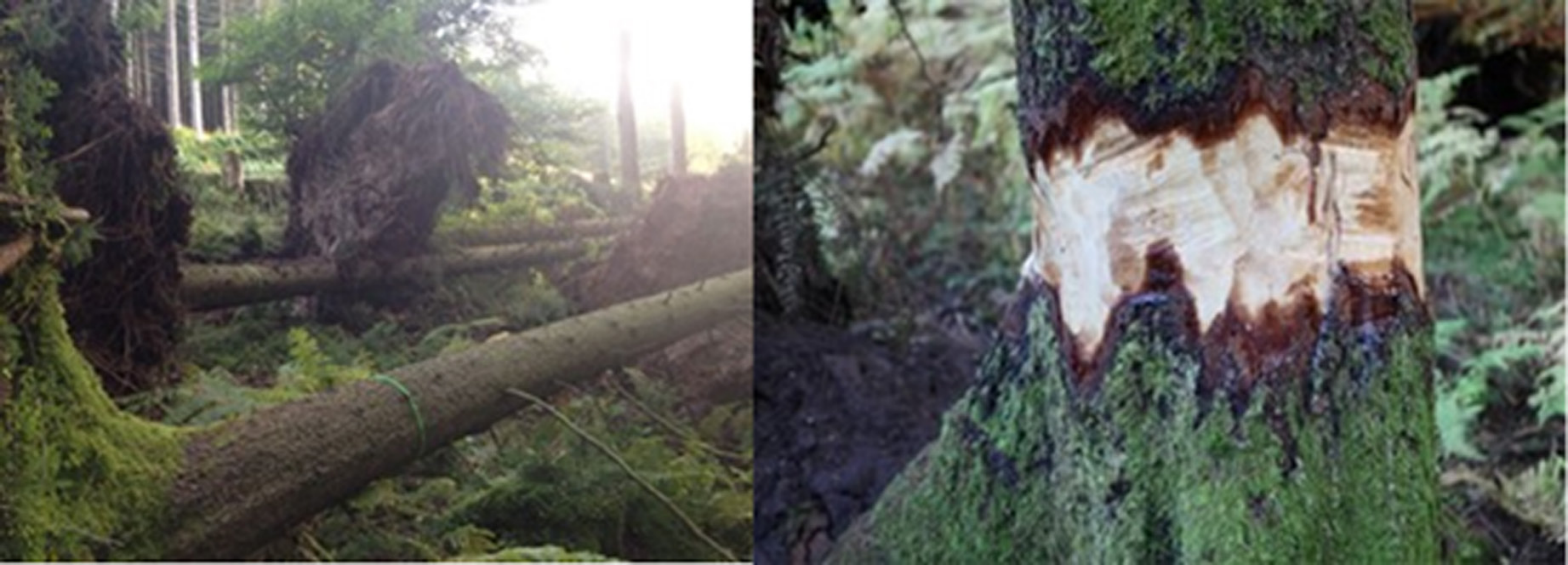
Structural complexity enhancement treatments to create downed and standing deadwood. Left is an example of the toppling and on the right an example of ring‐barking (girdling).

Care was taken to ensure that the six treatment plots matched the conditions of the six remaining control plots regarding exposition, slope, tree species composition, and stand structure. For each treatment plot, 10 trees were selected for each of the two treatments, comprising one randomly placed group of five trees and the remaining five trees randomly distributed across the plot area. The treatment of trees in groups was done to initiate the development of small gaps. We selected trees that had a similar DBH as the mean of all trees in a given plot. The trees selected for uprooting were toppled using a skidder with a winch. The trees were toppled in the direction the wind would have done in this valley to best mimic a natural disturbance (Figure 2). The other 10 trees were ringbarked at a height of around 60 cm from the ground with a chainsaw. Each bark‐free ring, around each tree had a width of about 40 cm to ensure that no cambium strips were left to connect the bark above and below the bark‐free ring (Figure 2).

The toppling and ring‐barking of selected trees was carried out in October 2016. On all 12 plots, x and y coordinates (in m) relative to the 0‐corner were determined by measuring distances from each tree to the x and y axes with a tape measure and an ultrasound rangefinder (TruPulse, Laser Technology Inc.). DBH (in cm) was measured for each tree of more than 15 cm DBH using a tape measure and all trees were numbered. For 10 trees per plot, tree height (in m) was also measured using a laser hypsometer (Vertex).

On all plots, standing, downed, and stump deadwood was inventoried before treatments. On all plots, standing deadwood, defined as all deadwood with a height > 1.3 m and at least 10 cm DBH, location, height, diameter at breast height, and decay stage were recorded. For all downed deadwood of at least 1.0 m in length and at least 10 cm in diameter at one end, diameters at both ends were recorded, as well as the x and y coordinates of each endpoint within the plot and the stage of its decay. For all stumps, defined as standing deadwood with a height of under 1.3 m, location, diameter at the cut surface, height, and state of decay were measured. Downed deadwood was measured only if it was within the limits of each plot, measuring the diameter at the plot border.

All TreMs relevant for the respective tree species were recorded for each tree following the TreM catalogue developed by Kraus et al. (2016), which lists 64 microhabitats for Central Europe. The TreMs include

- Cavities: woodpecker breeding cavities, rot holes, concavities, insect galleries, and bore holes;
- Tree injuries and exposed wood: exposed sapwood and/or exposed heartwood;
- Crown deadwood in different forms;
- Excrescences: twig tangles (witches' brooms), cankers, and burls;
- Fruiting bodies of saproxylic fungi and slime molds: perennial and ephemeral fungi fruiting bodies;
- Epiphytic, epixylic, and parasitic structures: epiphytic crypto‐ and phanerogams, nests of vertebrates and invertebrates, micro‐soil (i.e., resulting from decay of lichens, mosses or leaf litter in either thick old bark, or on horizontal limbs and forks for instance);
- Fresh exudates such as sap run and heavy resinosis.

The second inventory of trees, deadwood, and microhabitats took place from October 2020 to April 2021 and recorded all data for an analysis of the changes within the plots.

### 2.2 Statistical analyses

Tree volumes were calculated according to standard methods in forest mensuration, with stem volume being calculated as a cylinder and multiplied with a species‐specific form factor to correct for stem taper. These functions were taken from the literature (Bergel, 1973; Bergel & Bergel, 1974; Hillebrand, 1998; von Bergel, 1971).

Where such form factors were not available for the respective species, functions from similar tree species were used (see Appendix A). Some of the functions are for stemwood volume only, while others also estimate branchwood with a diameter above 7.0 cm. Where a function for stemwood with branches was available, we used that, but in most cases, only one function, usually stemwood, was available.

Volume was then calculated according to:

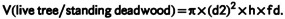




where d = DBH (cm), h = tree height (m), fd = form factor.

Standing deadwood volume was calculated using the same volume equations described above (form factor functions and volume calculation function).

Downed deadwood volumes were calculated using the formula for frusta (truncated cones), with length of the frustum derived from the positions of the endpoints, and dimensions from diameters at the endpoints.

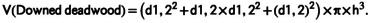




where d1, diameter 1; d2, diameter 2; h, length of deadwood section.

To analyze TreM abundance and richness per tree, we used generalized linear mixed models. To prevent autocorrelation (Dormann et al., 2007) in case trees within one plot might be more similar than between plots, we included a plot‐level factor and thus used generalized linear mixed models (GLMMs). The computation of the models took place in R (R Core Team, 2016). Since the abundance and richness data for TreMs were of count type, we built models with the glmmTMB function of the glmmTMB package (Bolker, 2016) with a Poisson distribution. In addition to assessing the control versus the treatment effect on the overall abundance and richness of TreMs per tree, we included the common predictor of DBH in the models (Asbeck, Großmann, et al., 2021).

$$\begin{array}{c} \text{Full}\;\text{model}:\text{TreM}\;\text{abundance}/\text{richness}\;\mathrm{per}\;\text{tree}‐\mathrm{DBH}\\ \;+\text{control}\;\mathrm{vs}.\text{treatment}+\left(1|\text{PlotID}\right). \end{array}$$

After model set up, we tested the simulated residuals of models with the Dharma package (Hartig, 2018). We used R‐packages ggplot2 (Wickham, 2009), lmfor (Mehtätalo & Lappi, 2020), and plyr (Wickham, 2011) as well as glmmTMB (Bolker, 2016).

## 3 RESULTS

### 3.1 Inventory results

**FIGURE 3:**
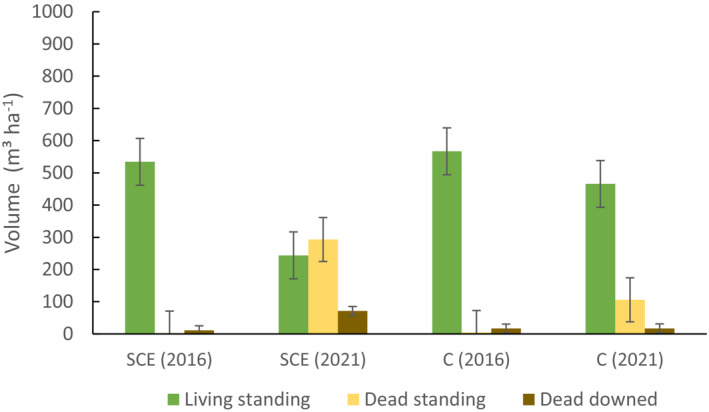
Volumes of living and dead standing wood as well as downed deadwood in the structural complexity enhancement treatment (SCE) and control (C) plots before the experiment (2016) and after 4 years (2021). The error bars indicate the standard deviation.

The results of the first inventory of stand structure before the SCE treatment showed a similar distribution of living and deadwood volumes in both the control and treated plots in 2016 (Figure 3; Table 1). Norway spruce dominated all plots. In 2021, following the measures taken that created a certain deadwood volume artificially, both downed and standing deadwood significantly increased in the experimental plots (Figure 3; Table 1). In two control plots, the standing deadwood increased significantly, whereas the downed deadwood volumes remained relatively similar (Figure 3; Table 1).

**TABLE 1:** Inventory results of the initial survey in 2016 and the repeated survey after the establishment of the experiment in 2020/21 in the treated (SCE, structural complexity enhancement) and control plots. BA, stand basal area.

Plot	Plot status	DBH (cm)			Tree species share of BA (%)			Volume (m^3^ ha^−1^)		
		Min.	Mean	Max.	Norway spruce	Other coniferous	Broadleaves	Dead standing	Living standing	Downed dead
**2016**										
1	SCE	21.9	44.1	67.0	92	2	6	0.0	647.4	6.1
3	SCE	15.6	37.0	62.7	90	8	2	4.2	670.3	5.2
6	SCE	11.9	33.8	59.8	100	0	0	2.2	428.0	5.9
7	SCE	15.0	36.0	68.0	97	1	3	0.3	508.3	6.5
8	SCE	16.8	42.4	73.5	100	0	0	0.0	495.0	17.5
10	SCE	15.4	33.7	57.4	84	11	5	10.0	456.4	25.5
2	Control	16.5	32.2	64.9	89	10	1	10.2	508.7	28.1
4	Control	15.1	33.8	59.3	74	26	0	4.0	741.9	14.8
5	Control	15.5	38.8	64.3	97	0	3	0.9	479.1	16.9
9	Control	15.1	32.5	79.0	88	12	0	3.9	582.6	7.2
11	Control	15.6	29.4	54.7	71	29	0	5.2	572.6	23.6
12	Control	17.8	36.0	52.5	76	17	8	3.2	516.4	8.4
**2021**										
1	SCE	16	42.8	69.5	90	2	8	237.6	409.8	94.4
3	SCE	15	35.8	64.0	85	12	3	589.7	84.7	56.5
6	SCE	15	36.7	60.0	100	0	0	95.0	335.3	50.4
7	SCE	15	37.2	68.5	95	1	4	335.9	172.7	66.6
8	SCE	18	44.7	75.0	100	0	0	411.8	83.2	88.8
10	SCE	17	34.2	67.0	82	11	7	88.3	377.9	69.8
2	Control	14	33.4	66.5	83	17	<1	174.9	344.0	31.9
4	Control	15	37.0	64.0	74	26	0	426.5	319.4	15.6
5	Control	16	39.5	66.0	98	0	2	0.0	480.0	11.8
9	Control	15	33.8	76.5	88	12	0	15.1	571.3	9.7
11	Control	15	31.0	55.0	69	31	<1	8.0	511.6	16.6
12	Control	15	36.0	64.0	76	16	8	10.8	566.9	16.7

### 3.2 Tree‐related microhabitat development between 2016 and 2021

**FIGURE 4:**
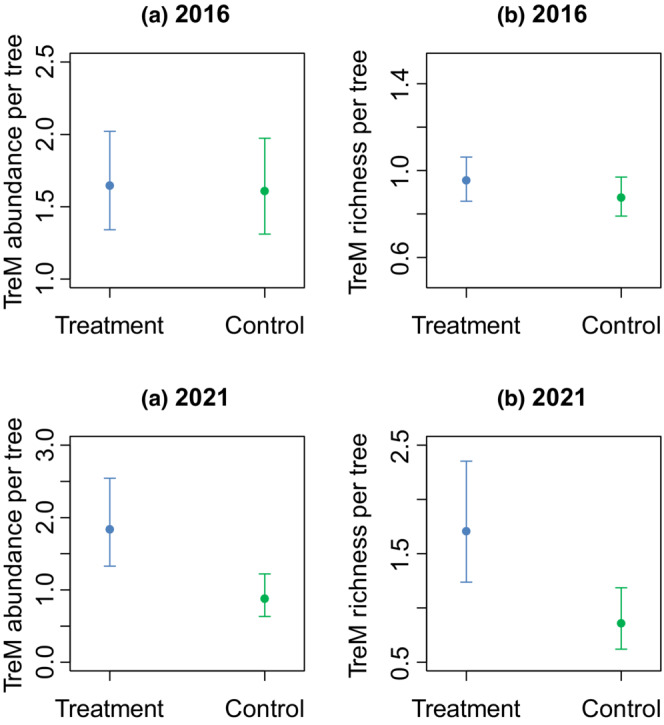
Results for the generalized mixed models for (a) TreM abundance and (b) TreM richness per tree in SCE treatment and control plots in 2016 (no significant difference) and 2021 (significant differences). The error bars indicate the 95% confidence interval.

An important change following the SCE treatment occurred in the abundance of TreMs in the control and experimental plots. The GLMMs showed no significant difference between both abundance and richness of TreMs in the control and experimental plots in 2016 (Table 2; Figure 4). Only DBH was a significant predictor of TreM abundance and richness. In contrast, there were significantly fewer TreMs in control plots compared to the experimental ones in 2021 (Table 2; Figure 4). The richness of TreMs per tree almost doubled in the experimental plots (1.6) compared to the control (0.8) in 2021.

**TABLE 2:** Results of the generalized linear mixed models for the abundance and richness of tree‐related microhabitats per tree indicating the estimate, standard error (SE), level of significance (*p*) for 2016 and 2021.

	2016				2021			
	Estimate	SE	*p*	Sign.	Estimate	SE	*p*	Sign.
TreM abundance								
Intercept	−0.46	0.13	<.001	***	−0.05	0.18	.78	
Plot status (control)	−0.02	0.15	.874		−0.74	0.24	<.01	**
DBH	0.03	0.00	<2 e‐16	***	0.02	0.00	<2 e‐16	***
TreM richness								
Intercept	−0.63	0.11	<.001	***	−0.06	0.18	.75	
Plot status (control)	−0.09	0.07	.248		−0.69	0.23	<.010	**
DBH	0.02	0.00	<.001	***	0.02	0.00	<2 e‐16	***

**FIGURE 5:**
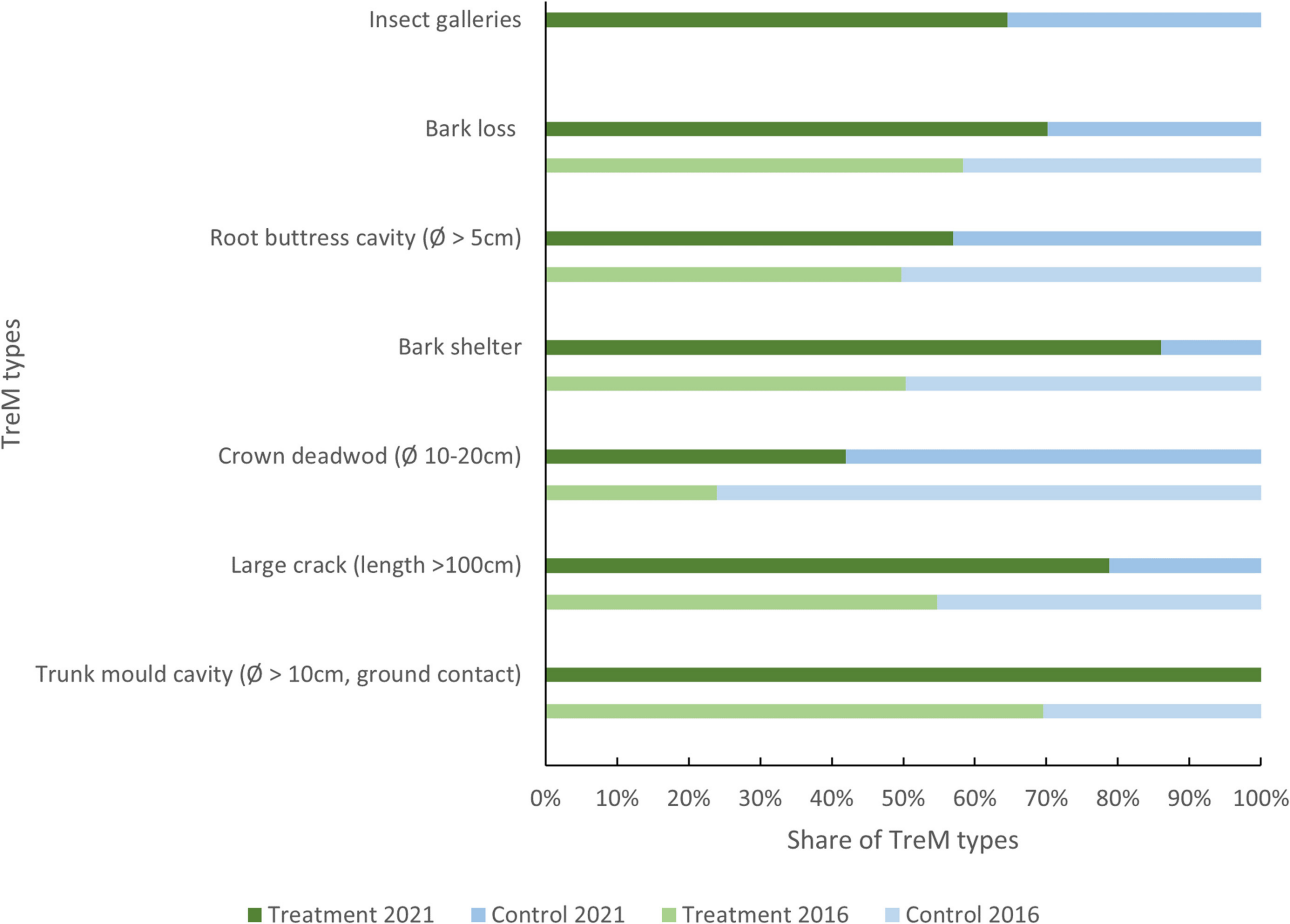
Share of selected types of tree‐related microhabitats following the description by Kraus et al. (2016) in the SCE treatment (green) and control (blue) plots inventoried in 2016 (upper bar) and 2021 (lower bar). The decreasing order follows the most frequent TreM types in 2021. Insect galleries were not present in 2016 (gray bar at the top), but were the most frequent TreM in 2021.

In total, we recorded 1795 TreMs consisting of 44 different types in the 2021 inventory (For supporting information see data accessibility statement). Owing to uncertainties due to sampling and observer bias, a direct comparison between 2016 and 2021 of absolute numbers for all TreM types, including rare ones, is not meaningful. Therefore, we provided an overview of selected TreM types, which were recorded in 2016 and 2021. The main TreM types were insect galleries, bark loss, and root buttress cavities (Figure 5). Many trees were recorded as being partially or completely without bark in the treatment plots in 2021 (Figure 5). Both the TreM type bark loss and the increase in insect galleries indicated that trees in the treatment plots faced somewhat different conditions that facilitated the formation of these TreMs, compared to those in the control plots. Only crown deadwood occurred more frequently in the control plots, whereas all other TreM types were more frequent in the treatment plots (Figure 5).

## 4 DISCUSSION

The enhancement of structural complexity through ring‐barking and toppling trees in Norway spruce dominated stands led to direct and indirect structural changes. Both deadwood volumes and tree‐related microhabitats increased significantly after the treatment.

Previous research that focused on the enhancement of structural complexity found that this increase in deadwood might be a positive change for the associated fungal community (Dove & Keeton, 2015). Moreover, the increase in deadwood is a typical goal when focusing on biodiversity relevant structures in forest ecosystems. For instance, in temperate mountain forests, minimum deadwood volumes of 30–40 m^3^ ha^−1^ have been recommended for the conservation of dead‐wood dependent species (Müller & Bütler, 2010). These threshold values have been clearly exceeded through the SCE treatment in the experimental plots. Especially for old‐growth indicator species, such as the three‐toed woodpecker, that is present in the research area, standing dead wood volumes of at least 15 m^3^ ha^−1^ have been recommended previously (Bütler et al., 2004). These volumes were artificially created through the SCE treatments in our experiment. The increase in deadwood is beneficial for many other species as reported previously (Eckelt et al., 2018; Müller et al., 2008). Forest management, as practiced in the Black Forest surrounding the national park and within the management zone of the national park, includes a constant removal of Norway spruce trees attacked by bark beetles to reduce the risk of further spread of bark beetles (Seidl et al., 2016). Yet, in terms of biodiversity conservation in a large and strictly protected area such as a national park, the salvage harvest of dead wood would be fundamentally opposing the biodiversity conservation goals (Thorn et al., 2018; Thorn, Seibold, et al., 2020).

Following the treatments, we can see a direct relation between the creation of standing and downed deadwood and an increase in insect galleries. This result, together with the increase in bark loss, underlines the increased biotic activity of saproxylic insects, notably *Ips typographus*, within the experimental plots, as links of this taxonomic group to the mentioned TreMs have been shown earlier (Basile et al., 2020; Paillet et al., 2018). Bark beetles therefore shape the habitat for many other species. In our experiment it was shown, for example, that wild bees benefit from the increased volume of dead standing trees (Eckerter et al., 2021). A second type of TreM that increased significantly after the treatment were bark shelters/pockets, which are an important habitat component as summer shelters for certain bat species (Basile et al., 2020; Parsons et al., 2003). These structures occurred frequently on standing decaying Norway spruce trees that slowly lose their bark. If TreMs are considered a multi‐taxon forest biodiversity indicator, their increase in abundance and richness suggests that the treated plots will host an increased species pool in the near future (Asbeck, Großmann, et al., 2021; Basile et al., 2020; Larrieu et al., 2018; Paillet et al., 2018).

Several limitations restrict extrapolation of the results of the experiment to similar situations. First, the treatment took place just before the severe drought events of 2018 and 2019 in an area where especially Norway spruce is susceptible to drought stress (Schuldt et al., 2020; Vitali et al., 2017). Therefore, the significant increase in deadwood volumes is not solely attributable to the treatment itself, but likely also to the spread of bark beetles from the artificially killed trees to the surrounding stands owing to favorable conditions for beetles in drought‐stressed trees. Hence, the deadwood dynamics may be quite different, if such treatments were followed by cool and wet years. Yet, the experiment shows that these SCE treatments have the potential to trigger a local bark beetle outbreak. However, the effect of a high bark beetle population will decline after the natural cycle has peaked and their numbers decrease. A second limitation regarding the TreM inventory accuracy is the strong possibility of observer bias (Paillet et al., 2015). There are almost no time series data of TreM development available based on empirical data (Puverel et al., 2019), just a single study reported the cross‐sectional development of TreMs (Courbaud et al., 2017), probably grounded in the differences existing (observer bias) in TreM inventories. Even when using a standardized and easy‐to‐follow inventory protocol (Kraus et al., 2016; Larrieu et al., 2018), the results showed relatively large differences in the recording of TreMs for individual trees in control plots. This may be partially explained by the significant mortality, which changes tree attributes and also the visibility of TreMs in defoliated crowns. If repeated inventories of the same trees are carried out, it is crucial to streamline the recording methodology of TreMs beforehand. Importantly, in our case, even after doing so, we could not entirely compare the TreM results of 2016 to those of 2020. This is due to the fact that several categories of TreMs need to be estimated by the inventory team, for instance the percent cover of lichens. Nevertheless, between the treatment and control plots of each inventory, the inventory and analyses were consistent and robust enough to yield clear results.

## 5 CONCLUSIONS

Overall, the SCE treatment successfully increased biodiversity relevant structures in Norway spruce‐dominated stands in the Black Forest National Park previously managed for wood production. Especially the volume of deadwood as well as the abundance and richness of tree‐related microhabitats, which can support a large array of taxonomic groups, increased significantly. This result indicates that the treatments are suited to actively restore some structural features of old‐growth forests. The next steps would be to quantify the biodiversity response of our treatments on other taxonomic groups such as birds, bats, small mammals, saproxylic insects, fungi, and the ground vegetation.

## AUTHOR CONTRIBUTIONS

**Thomas Asbeck:** Data curation (lead); formal analysis (lead); investigation (equal); methodology (equal); supervision (supporting); visualization (lead); writing – original draft (lead); writing – review and editing (lead). **Adam Benneter:** Data curation (equal); investigation (equal); methodology (equal); resources (equal); writing – original draft (equal); writing – review and editing (supporting). **Alexander Huber:** Data curation (supporting); investigation (supporting); writing – original draft (supporting). **Damaris Margaritis:** Investigation (equal); methodology (supporting); project administration (equal); writing – original draft (equal); writing – review and editing (equal). **Joern Buse:** Conceptualization (supporting); data curation (supporting); funding acquisition (supporting); investigation (supporting); methodology (supporting); writing – original draft (supporting); writing – review and editing (supporting). **Flavius Popa:** Formal analysis (supporting); investigation (equal); methodology (supporting); writing – original draft (supporting); writing – review and editing (supporting). **Patrick Pyttel:** Conceptualization (supporting); data curation (equal); formal analysis (supporting); investigation (supporting); methodology (supporting); project administration (supporting); supervision (supporting); writing – original draft (supporting); writing – review and editing (supporting). **Marc Förschler:** Conceptualization (supporting); funding acquisition (equal); investigation (equal); methodology (equal); project administration (supporting); resources (equal); supervision (equal); writing – original draft (equal); writing – review and editing (equal). **Stefanie Gärtner:** Conceptualization (equal); data curation (equal); funding acquisition (supporting); investigation (equal); methodology (equal); project administration (equal); writing – original draft (equal); writing – review and editing (equal). **Jürgen Bauhus:** Conceptualization (lead); funding acquisition (lead); methodology (lead); project administration (lead); supervision (lead); writing – original draft (equal); writing – review and editing (equal).

## CONFLICT OF INTEREST

No competing interests.

## Data Availability

Locations of plots and individual tree information including tree related microhabitats will be published in Dryad: https://datadryad.org/stash/share/tl_QkSDnvbPpzZMrK31hc3HcrEwlK10pixYdlxKDkjM.

## APPENDIX A

The form factor functions used here were:

1. *Picea abie*s = 0.5848 + 3.34262/(d2) − 1.73375/(d × h) − 0.26215 × ln(h)/ln(10.0) + 0.18736 × ln(d)/ln(10.0) + 11.34436/(h × d2)
2. *Pinus sylvestris/strobus* = 0.35096 + 0.93964/h + 1.5464/d  − 2.0482/(h × h) − 5.7305/(h × d) + 17.444/(d × h2)
3. *Pinus strobus* = 0.35096 + 0.93964/h + 1.5464/d − 2.0482/(h × h) − 5.7305/(h × d) + 17.444/(d × h2)
4. *Larix decidua* = −0.583 + 4.52132/(d × d) − 5.59827/(d × h) − 0.2101 × ln(h)/ln(10.0) + 0.12363 × ln(d)/ln(10.0) + 21.92938/(h × d2)
5. *Fagus sylvatica/Betula pendula/Ilex aquifolia* = 0.4039 + 0.0017335 × d + 1.1267/d − 118.188/(h × h × h) + 0.0000042 × h2.
6. *Pseudotsuga menziesii* = −200.31914/(d × h2) + 0.8734/h − 0.0052 × LN(h)2 + 7.3594/(d × h) + 0.46155.
7. *Quercus rubra* = 0.4237 + 0.039178/h − 4.69154/(h2) + 38.5469/(d × h) − 335.8731/(d × h2)
8. *Sorbus aucuparia* = 0.4786 − (1.011176/h) + (2.10428/d) − (203.1997/(h × d2))

where d = DBH (cm) and h = height (m).
